# Use of textural analysis of tumour vascular heterogeneity as a biomarker of response to anti-angiogenic treatment in patients with nasopharyngeal carcinoma

**DOI:** 10.1186/1470-7330-14-S1-P23

**Published:** 2014-10-09

**Authors:** Qiao Qi Teo, Benjamin Haaland, Choon Hua Thng, Wan Teck Lim, Tong San Koh, Quan-Sing Ng

**Affiliations:** 1 Duke-NUS Graduate Medical School, Singapore, Singapore; 2 Department of Oncologic Imaging, National Cancer Centre Singapore, Singapore, Republic of Singapore; 3 Department of Medical Oncology, National Cancer Centre Singapore, Singapore, Republic of Singapore

## Objectives

This study aims to evaluate the impact of tumour vascular heterogeneity, derived from textural analysis of dynamic contrast-enhanced computed tomography (DCE-CT) perfusion characteristics in patients with NPC treated with the anti-angiogenic drug, pazopanib.

## Materials and methods

DCE-CT images of 33 patients, with recurrent/metastatic NPC treated with pazopanib, were analyzed to derive tumour blood flow, fractional intravascular blood volume, fractional extracellular-extravascular volume, and permeability surface area product. To each parametric map, textural analysis using multiple parameters based on co-occurrence matrix, neighbourhood gray tone difference, run-length matrix, gray level size-zone matrix and histogram statistics were applied to characterize its heterogeneity. Reproducible and important textures, were selected by a cut-off on the coefficient of variation and variable importance determined by random forest analysis, respectively. These textures are summarized by principal components analysis and their relationship to outcomes evaluated using Cox proportional hazards, receiver-operating characteristics, Kaplan-Meier and logistics regression analysis.

## Results

The first principal component of the reproducible and important textural features of pre-treatment fractional intravascular blood volume (PC1) was related to survival outcomes. Using Kaplan Meier analysis, overall survival was significantly higher for patients with PC1 values greater than the median (p=0.002). There was no statistically significant association between DCE-textures and overall response.

## Conclusion

Heterogeneity of tumour intravascular blood volumes may be an important biomarker for predicting overall survival in patients with NPC treated with anti-angiogenic therapy.

